# Supplementary material for: A 2-Gene Host Signature for Improved Accuracy of COVID-19 Diagnosis Agnostic to Viral Variants
Source: mSystems. 2022 Dec 12;8(1):e00671-22. doi: 10.1128/msystems.00671-22 (PMC9948727; doi:10.1128/msystems.00671-22)
Supplement: TABLE S1 [file msystems.00671-22-s0002.docx]

**Supplementary Table 1.** Cohort details.

| **Cohort** | **Description** | **COVID-19 (n)** | **Non-Viral ARI (n)** | **Other Viral ARI (n)** | **Reference** |
| --- | --- | --- | --- | --- | --- |
| 1. UCSF (RNA-seq, n=318) | Patients tested for COVID-19, CA, 2020 | 90 | 169 | 59 | ^10^ + this study |
| 2. New York (RNA-seq, n=553) | Patients tested for COVID-19, NY, 2020 | 166 | 308 | 79 | ^12^ |
| 3. UCSF (qPCR, n=144) | Patients tested for COVID-19, CA, 2020 | 72 | 72 | | This study |
| 4. UCSF SARS-CoV-2 N-gene variant (qPCR, n=4) | Patients with SARS-CoV-2 N-gene variant, CA, 2020 | 4 | - | | This study |
| 5. UCSF SARS-CoV-2 Delta variant (qPCR, n=7) | Patients with SARS-CoV-2 Delta variant, CA, 2021 | 7 | - | | This study |
| 6. UCSF SARS-CoV-2 Omicron variant (qPCR, n=3) | Patients with SARS-CoV-2 Omicron variant, CA, 2021 | 3 | - | | This study |
