# Supplementary figures and images for: A 2-Gene Host Signature for Improved Accuracy of COVID-19 Diagnosis Agnostic to Viral Variants
Source: mSystems. 2022 Dec 12;8(1):e00671-22. doi: 10.1128/msystems.00671-22 (PMC9948727; doi:10.1128/msystems.00671-22)

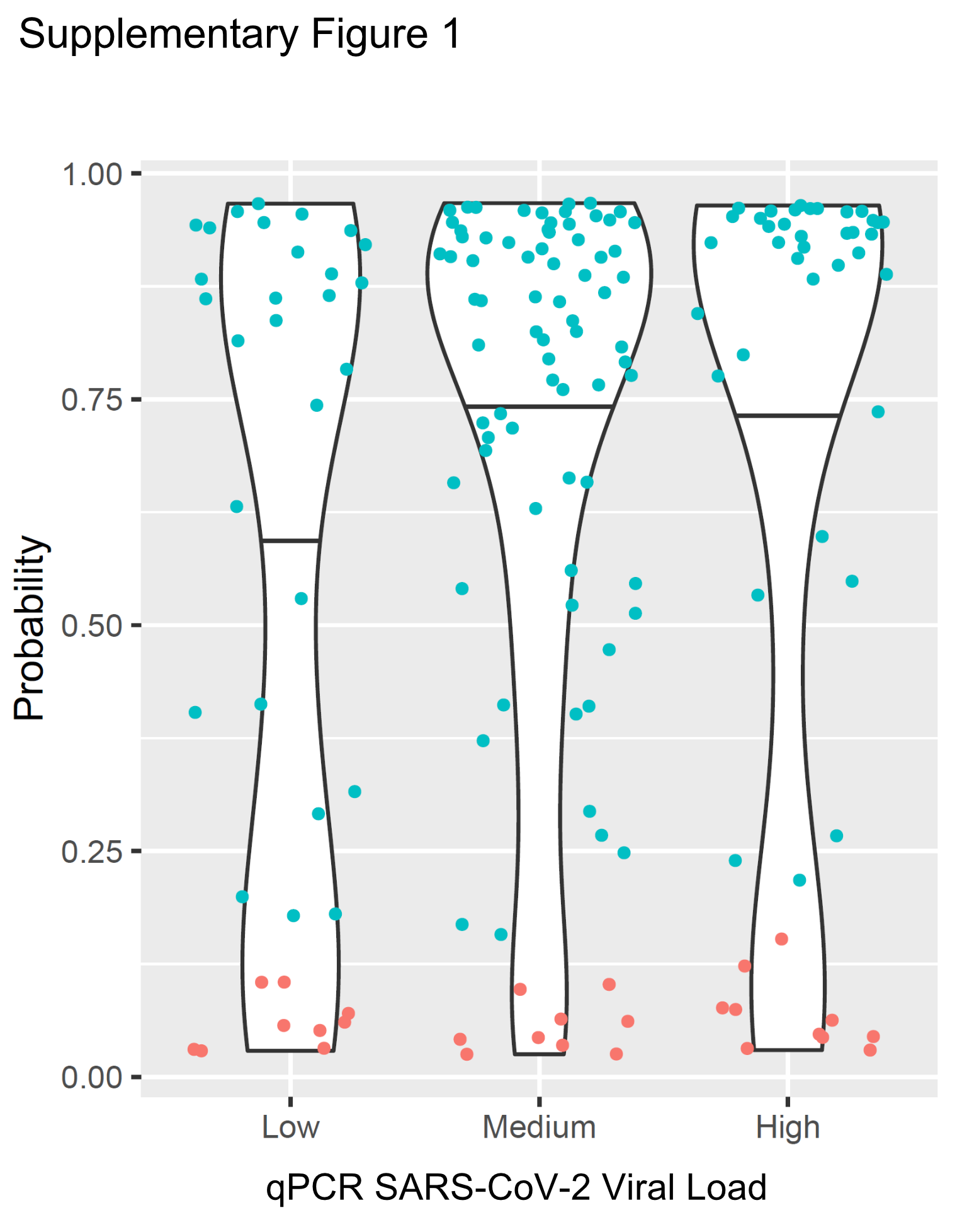

Supplement: FIG S1 [file msystems.00671-22-s0001.tif]
