# Supplementary material for: A 2-Gene Host Signature for Improved Accuracy of COVID-19 Diagnosis Agnostic to Viral Variants
Source: mSystems. 2022 Dec 12;8(1):e00671-22. doi: 10.1128/msystems.00671-22 (PMC9948727; doi:10.1128/msystems.00671-22)
Supplement: TABLE S2 [file msystems.00671-22-s0003.docx]

**Supplementary Table 2.** Taqman Gene Expression assay IDs for genes tested by RT-qPCR.

| **Gene** | **Assay ID** |
| --- | --- |
| *IFI6* | Hs00242571_m1 |
| *GBP5* | Hs00369472_m1 |
| *RPP30* | Hs01124518_m1 |
