# Supplementary material for: A 2-Gene Host Signature for Improved Accuracy of COVID-19 Diagnosis Agnostic to Viral Variants
Source: mSystems. 2022 Dec 12;8(1):e00671-22. doi: 10.1128/msystems.00671-22 (PMC9948727; doi:10.1128/msystems.00671-22)
Supplement: TABLE S3 [file msystems.00671-22-s0004.docx]

**Supplementary Table 3.** Performance characteristics of the *IFI6*+*GBP5* COVID-19 diagnostic classifiers in qPCR assays. The positive predictive value (PPV), negative predictive value (NPV), sensitivity (Sens), and specificity (Spec) values shown were calculated at Youden’s index and aggregated across the five cross-validation folds.

| **Metric** | **Value** |
| --- | --- |
| PPV | 0.926 |
| NPV | 0.753 |
| Sens | 0.694 |
| Spec | 0.944 |
